# Beneficial Effects of Spermidine on Ovarian Function, Gut Microbiota Composition, and Associated Metabolic Changes

**DOI:** 10.3390/nu18121874

**Published:** 2026-06-10

**Authors:** Chengweng Ji, Dongmei Jiang, Yunxuan Wu, Jue Huang, Yuxin Qi, Xin Wang, Weijie Zhang, Shuo Li, Lu Lu, Mingzhou Li, Bo Kang

**Affiliations:** State Key Laboratory of Swine and Poultry Breeding Industry, College of Animal Science and Technology, Sichuan Agricultural University, Chengdu 611130, China

**Keywords:** spermidine, ovarian function, gut microbes, metabolomics

## Abstract

Background: Spermidine is involved in a wide range of cellular processes, including mammalian oocyte development. Wheat germ is a natural source of polyamines and contains high concentrations of spermidine. However, no studies have evaluated the effects of wheat germ-derived spermidine on the regulation of mammalian ovarian function. The present study aimed to investigate the effect and underlying mechanism of wheat germ-derived spermidine on mammalian ovarian function. Methods: A feeding trial was carried out on mice with diets supplemented with varying concentrations of spermidine. The underlying mechanism by which spermidine exerts its beneficial effects on ovarian function and fertility in mice was explored through the integration of serum metabolomics and intestinal microbiomics analyses. Results: The results showed that dietary spermidine-rich feed significantly increased spermidine absorption and affected the metabolism of spermine and putrescine in the intestines. Dietary intake of low-concentration spermidine significantly increased the number of pups per litter and the secretion levels of estradiol (E2), progesterone (P4), luteinizing hormone (LH), and anti-Müllerian hormone (AMH). Furthermore, compared with a normal diet, spermidine supplementation resulted in significantly higher ovarian reserves and fewer atretic follicles. Correspondingly, metabolomics analysis revealed that spermidine primarily affected lipid metabolism and endocrine functions related to reproduction. In addition, dietary spermidine significantly altered the structural composition of the gut microbiota. Correlation analysis demonstrated that the abundance of *Oceanisphaera*, *Atopostipes*, and *Actinobacteriota* was significantly positively correlated with the secretion of E2, P4, and LH. Conclusions: Overall, these findings yield phenotypic insights into spermidine’s mediation of mammalian reproductive performance and offer a potential therapeutic strategy for individuals with reproductive dysfunction.

## 1. Introduction

Genetic elements, environmental influences, autoimmune disorders, surgical procedures, and certain drug therapies are risk factors for the loss of ovarian function [1]. Primary ovarian insufficiency, in which women below the age of 40 have ovarian follicles that may be depleted or become dysfunctional, affects approximately 1% of women in their childbearing years [2]. Spermidine, a natural inducer of autophagy, is widely present in eukaryotic cells and plays indispensable roles in cell proliferation, growth, and metabolism [3,4]. The abundance of spermidine declines during the aging process of organisms, and is regarded as a well-tolerated mimic of caloric restriction that resists a variety of molecular and physiological age-related adverse effects [3]. Due to its autophagy-inducing and anti-aging properties, spermidine’s roles in age-associated chronic diseases (including cardiovascular disorders and neurodegenerative conditions) and cancers have become a focus of research, with aging models frequently employed in spermidine studies [5,6]. Notably, accumulating studies in recent years have indicated that spermidine exerts beneficial effects on female reproductive processes, and it is essential for female reproduction as well as embryonic and fetal development. Spermidine deficiency can result in embryonic developmental arrest, whereas exogenous spermidine supplementation alleviates such arrest by mitigating oxidative stress [7,8]. Additionally, spermidine has been demonstrated to ameliorate, via multiple molecular mechanisms, pathological damage to rat ovarian tissues induced by diabetes. Specifically, it enhances the antioxidant defense capacity of tissues and reduces MDA levels, while upregulating the expression of LC3 and Beclin-1, thereby markedly promoting autophagic activity [9]. Consistently, relevant investigations on ovarian oocytes and granulosa cells further confirm that spermidine maintains cellular quality and physiological functions mainly through multiple pathways, including strengthening cellular antioxidant defense, inhibiting cell apoptosis, inducing autophagy, and improving mitochondrial function [10,11,12,13,14,15].

Numerous studies have indicated that intestinal commensal bacteria are important regulators of physiological homeostasis within the body [16], thus influencing ovarian function and reproductive performance in animals [17,18,19,20]. These bacteria substantially affect both human health and disease [21]. Structural alterations in the gut microbiota have been found to interact with various reproductive disorders, such as polycystic ovary syndrome [22,23], endometriosis [24,25], and cervical cancer [26,27]. Furthermore, a strong link exists between gut microbes and steroid hormones [28,29,30,31]. A recent study has found a highly relevant link between differential microbes and steroid hormone secretion levels in highly and poorly bred sows [32]. Notably, spermidine is an important metabolite product of gut microbes that not only promotes the growth and proliferation of intestinal epithelial cells [33,34], but also exerts important impacts on the gut microbiota’s structural composition [35,36]. Many studies have shown that spermidine has positive therapeutic effects in colitis models by facilitating the proliferation and growth of intestinal epithelial cells, and accelerating the repair of damaged intestinal tissue [37,38]. Different gut microbial communities have varying effects on the intestinal environment and can influence distal organs and pathways. The gut microbiota is considered a fully functional endocrine organ [39]. Accumulating evidence has demonstrated that spermidine exerts beneficial effects on both the regulation of intestinal microbiota and reproductive performance in mammals. Nevertheless, whether the beneficial effects of spermidine on intestinal microbiota are correlated with animal reproductive performance remains unclear.

Wheat germ, one of the main by-products of wheat during food processing and a source of important nutritional value, is a natural source of polyamines, particularly spermidine. Several studies have found that wheat germ is the plant source with the highest spermidine concentration, which can reach 354 mg/kg. Existing extraction techniques can concentrate spermidine in wheat germ to tens of times its original concentration. Furthermore, studies on wheat germ-derived spermidine have confirmed its favorable biosafety in animals and revealed its potential to exert beneficial physiological effects [40,41]. Most current studies on spermidine have used chemically synthesized spermidine. Although most of those studies have shown satisfactory results, chemically synthesized spermidine persistently poses concerns regarding dosage safety [42,43], which requires prolonged investigation, whereas plant-derived natural spermidine offers a novel potential avenue for research and application.

Therefore, conducting studies on the transformation of spermidine is challenging. Using wheat germ spermidine might provide a novel approach to facilitate studies on spermidine transformation. Moreover, current research on wheat germ spermidine supports the exploration of higher concentrations or longer treatment durations [41]. The study of interactions between host health and gut microbes is highly challenging. Metabolites, serving as mediators between the two, are a crucial means of investigating gut microbial function [44]. Therefore, in this research, the effects of wheat germ spermidine on the reproductive performance of mice fed diets containing different proportions of wheat germ extract (1% spermidine) in vivo were evaluated by measuring fertility and changes in ovarian function. The relationship between spermidine-induced changes in the gut microbiota and ovarian function was examined through a microbiomics and metabolomics approach.

## 2. Materials and Methods

### 2.1. Animal Ethics Statement

All feeding procedures in this experiment were approved by the Institutional Animal Care and Use Committee of Sichuan Agricultural University (Chengdu Campus, Chengdu, China), under Approval No. 20240142. Experimental procedures also comply with the Guide for the Care and Use of Laboratory Animals of the National Institutes of Health.

### 2.2. Diet Preparation and Animal Feeding

**Figure 1:**
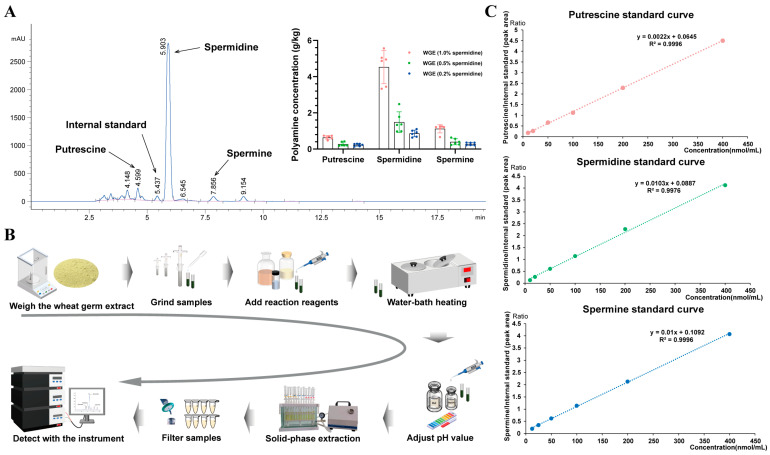
Determination and analysis of polyamine contents in wheat germ extract. (**A**) Procedure for the measurement of polyamine content in wheat germ extract. (**B**) Actual polyamine detection results of extracts containing different concentrations of spermidine (1%, 0.5% and 0.2%) (n = 6). (**C**) Construction of standard curve for polyamines (putrescine, spermidine and spermine). WGE: Wheat germ extract.

The spermidine-rich wheat germ extract used in this study was purchased from Focus Herb Co., Ltd. (Xi’an, China). Based on our previously established polyamine determination method [45], we analyzed the polyamine concentrations of three wheat germ extracts containing different concentrations of spermidine, and the detection results are shown in Figure 1A. The assay procedure and the corresponding standard curves for polyamine determination (n = 6) are shown in Figure 1B,C. Compared with other polyamines, spermidine was the most dominant component in wheat germ extract, which supported our further study. Feed formulation was designed by Chengdu Dossy Experimental Animals Co., Ltd. (Chengdu, China), and the specific feed composition is presented in Table 1. The experimental animals included 140 6-week-old C57BL/6 female mice purchased from Chengdu Dossy Experimental Animals Co., Ltd. (Chengdu, China). The number of mice was determined using the error degrees of freedom (E) from analysis of variance. With 4 groups and 35 female mice per group (total n = 140), E = 140 − 4 = 136, which exceeds the recommended minimum and ensures sufficient statistical power. The number of additional mice beyond this minimum was estimated based on the sample size required for indicator measurements throughout the experiment. Schwarz et al. suggested that future studies on wheat germ-derived spermidine should explore its biological functions at higher concentrations. Therefore, based on previous research, we increased the experimental concentrations and established three gradients [41]. Female mice were divided into four groups: a 0 g/kg wheat germ extract group (Control), 50 g/kg wheat germ extract group (L SPD), 100 g/kg wheat germ extract group (M SPD), and 150 g/kg wheat germ extract group (H SPD) (n = 35). According to our actual detection results, these groups corresponded to mice’s daily spermidine intake of 0 mg/kg, 70 mg/kg, 140 mg/kg, and 210 mg/kg body weight (n = 35). Each group comprised seven cages of mice, with five mice per cage. All mice were pre-fed 1 week in advance. The feed was then changed to spermidine feed, and the mice were fed for 12 weeks with food and water provided ad libitum. All mice used in the feeding experiment were obtained from the same batch and randomly assigned to cages. Animals from different groups were placed alternately within the same rearing environment. Diet renewal, water replacement, bedding change and phenotypic data collection were conducted at a fixed time every week. All mice were euthanized via inhalation of 4% isoflurane in accordance with the Guide for the Care and Use of Laboratory Animals of the National Institutes of Health. Following euthanasia, intestinal tissues, ovaries, cecal contents, and serum samples were collected immediately. All samples were rapidly frozen in liquid nitrogen and stored at –80 °C for subsequent analysis.

### 2.3. Fertility Experiments

A control group and three groups with spermidine concentration gradients were established. Each group comprised 14 female and 7 male mice, all of which were 6-week-old C57BL/6J mice. Female and male mice were co-housed for mating at a female-to-male ratio of 2:1. After 12–14 h of co-housing, successful mating was confirmed by observing the presence of a vaginal plug in female mice. The plugged females were then housed individually, and pregnancy was further confirmed by monitoring external phenotypic signs. On gestational day 20, the number of pups per litter was recorded for each pregnant female in each group. Following a 7-day rest period, the same females were re-paired with males for another round of mating. The entire reproductive experiment lasted for three months, during which the average litter size per female in each group was calculated to assess the differences in female fertility among the treatment groups.

### 2.4. Estrous Cycle Monitoring

Changes in the estrous cycle of mice were observed according to vaginal smears (n = 5). Dried smears were stained with hematoxylin–eosin. The estrous phase was then determined according to the morphological characteristics of each estrous cycle. Two trained laboratory professionals initially examined the vaginal smears to identify the estrous phase. If they could not reach agreement, a third technician conducted the observation. The stages were characterized by the following morphological features. In proestrus, the predominant cells were nucleated epithelial cells. The nuclei of these cells stained purple, the cytoplasm was rose-red, and the cell membranes were intact. In estrus, primarily rose-red nucleated keratinized cells with irregular shapes were observed, piled in layers. The smears contained a high density of cells filling the entire field of view. In metestrus, a substantial number of leukocytes and anucleate keratinized epithelial cells were present. These cells were round or oval, and demonstrated dark staining. In diestrus, all three types of cells—nucleated epithelial cells, keratinized cells, and leukocytes—were present at an average cell density.

### 2.5. Determination of Reproduction-Associated Hormones in Serum

After the 90-day feeding period, blood was collected from the retrobulbar intraorbital capillary plexus of the mice under 2% isoflurane anesthesia. Collected mouse blood was placed in 1.5 mL centrifuge tubes, and sera were separated off through centrifugation at 3500 rpm for 15 min. Serum levels of reproduction-associated hormones (E2, P4, FSH, LH, and AMH) were measured with an ELISA kit according to the manufacturer’s instructions (Jiangsu Meimian Industrial Co., Ltd., Yancheng, China) (n = 8). In brief, the kit was removed from 4 °C and allowed to equilibrate at room temperature for 20 min. Standard and sample wells were prepared by adding 50 μL of standards with different concentrations to standard wells, 10 μL of test samples to sample wells (followed by dilution with 40 μL sample buffer), and leaving blank wells unoccupied; 100 μL of horseradish peroxidase-conjugated detection antibody was then added to all standard and sample wells (not blank wells), after which reaction wells were sealed with a plate-sealing membrane and incubated at 37 °C in a thermostat for 60 min. The liquid was discarded, wells blotted dry on absorbent paper, and each well filled with washing buffer (allowed to stand for 1 min) before the buffer was decanted and wells blotted dry—this washing step was repeated five times. Next, 50 μL each of Substrate A and B was added to all wells for 15-min incubation at 37 °C in the dark, followed by 50 μL stop solution per well; finally, the optical density (OD) of each well was measured at 450 nm, and serum hormone concentrations were calculated using sample OD values and the standard curve equation.

### 2.6. Reverse Transcription Quantitative PCR

Total RNA was extracted using the Trizol method, and reverse transcription was conducted in accordance with the manufacturer’s protocols and instructions for the PrimeScript RT reagent Kit (with gDNA Eraser), provided by Takara Bio Technology Co., Ltd. (Dalian, China). The sequences of primers and lengths of amplification products are presented in the primer information table (Table 2). Per the guidelines outlined in the TB GreenTM Premix Ex TaqTM II kit, a 10 μL reaction system was prepared for use in the RT-qPCR assay, using cDNA as the template. This system included 5 μL of TB Green Premix Ex Taq II, 0.2 μL of forward primer, 0.2 μL of reverse primer, 1 μL of cDNA, and 3.6 μL of RNase-Free dH2O. The RT-qPCR reaction was carried out under the following parameters: 95 °C for 30 s, 60 °C for 30 s, and 72 °C for 30 s, with a total of 35 cycles. After amplification, both amplification curves and melting curves were generated, and CT values were calculated for subsequent result analysis (n = 6).

### 2.7. Polyamine Determination

Under liquid nitrogen, tissue samples weighing 0.05–0.15 g were collected and placed in centrifuge tubes (n = 6). Subsequently, 1 mL 5% HClO_4_ and 10 μL internal standard working solution (1,6-hexanediamine) were added. The samples were then transferred to a glass homogenizer and ground to ensure thorough mixing of the internal standard working solution and the samples. Afterward, the samples were transferred to a new centrifuge tube (while avoiding light). The samples were vortexed and shaken to achieve good mixing, then crushed with an ultrasonic cell crusher for 10–15 min. Centrifugation was performed at 8500–9000 rpm for 10 min, and the supernatant was transferred to a new 15 mL centrifuge tube. To the centrifuge tube containing the precipitate, 1 mL 5% HClO_4_ was added, vortexed, and shaken to mix well; this was followed by centrifugation and transfer to the same centrifuge tube. Subsequently, enough 2.5 mol/L NaOH and 12 μL benzoyl chloride were added, and the mixture was placed in a 40 °C water bath for derivatization for 1 h. The pH of the derivatization solution was adjusted to approximately 7.0 with 2.5 mol/L NaOH and 6 mol/L HCl solution. The samples were then transferred to a C18 solid phase extraction column for extraction. After extraction, impurities were washed away with ultrapure water and 15% methanol. Finally, the samples were eluted with 500 μL 100% methanol for HPLC detection. The parameters were as follows: mobile phase, methanol:water = 62%:38%; flow rate, 1 mL/min; injection volume, 20 μL; detection time, 20 min/sample; UV detection wavelength, 229 nm; and column temperature, 40 °C. Subsequently, polyamine concentration in the sample solution (nmol/mL) was calculated based on the detection results and a standard curve equation for polyamines. This standard curve was generated by replacing the sample with standard solutions at various concentrations while keeping all other steps identical to those used for sample detection. Finally, total polyamine content in the tissue (mg/kg) was calculated using the sample mass recorded at the initial weighing.

### 2.8. HE Staining and Follicle Count

Left ovaries of mice were fixed in 4% paraformaldehyde for 24 h. After dehydration, the ovaries were routinely embedded in paraffin. Subsequently, the embedded ovaries were serially sliced at a thickness of 5 μm, and one slice was taken at every five intervals for hematoxylin–eosin staining. A digital slice scanner was used to observe the ovarian slices. Follicles were sorted microscopically and in the mouse ovarian tissues, primordial follicles, primary follicles, secondary follicles, and atretic follicles were manually counted (n = 4). The work on ovary slices and the follicle counting were performed by Chengdu Lilai Biotechnology Co., Ltd. (Chengdu, China).

### 2.9. 16s rRNA Sequencing

After 90 days of spermidine feeding, cecal contents were collected from mice, promptly harvested, and snap-frozen in liquid nitrogen for DNA extraction (n = 6). DNA quality/concentration was assessed via 1% agarose gel electrophoresis and NanoDrop 2000 (Thermo Scientific, Waltham, MA, USA), and then samples were stored at −80 °C. Subsequently, the bacterial 16S rRNA gene V3–V4 region was PCR-amplified with primers 338F (5′-ACTCCTACGGGAGGCAGCAG-3′) and 806R (5′-GGACTACHVGGGTWTCTAAT-3′); PCR products were retrieved from 2% agarose gel, purified with Major Bio’s DNA Gel Recovery Kit, and quantified via Qubit 4.0 (Thermo Fisher, Waltham, MA, USA). Sequencing was done on Illumina PE300/PE250 (Major Bio, Shanghai, China); raw reads underwent QC with fastp (v0.19.6), assembled via FLASH (v1.2.11), and post-QC optimized sequences were denoised via DADA2 plugin in Qiime2. Based on the results of ASV clustering analysis, diversity index analysis was performed, and sequencing depth was detected. Finally, a series of in-depth statistical and visualization analyses of community structure and phylogeny were conducted in R language (Version 4.4.0).

### 2.10. Non-Targeted Metabolomics

Prior to euthanasia, blood was collected from anesthetized mice’s hearts via a 1-mL syringe (n = 6). The blood was then centrifuged at 4 °C (3500 rpm, 15 min) to isolate serum for LC-MS analysis. Thermo Fisher’s UPLC-FT-MS served as the metabolomic platform. For LC-MS data, raw files underwent baseline filtering, peak detection, and retention time alignment via Progenesis QI (Waters); mass spectral data were matched to HMDB/Metlin databases for metabolite info. Principal component analysis (PCA) and orthogonal partial least squares-discriminant analysis (OPLS-DA) were run via R package ropls (v1.6.2). Student’s *t*-tests and differential metabolite screening were conducted, with selection based on OPLS-DA-derived VIP scores and *t*-test *p*-values; metabolites with VIP > 1 and *p* < 0.05 were defined as differential.

### 2.11. Data Analysis

The Shapiro–Wilk test was first used to assess the normality of all data. After confirming normality, the data are presented as the mean ± standard error (SEM). Statistical analysis was performed using one-way analysis of variance (ANOVA) and Student’s *t*-tests in SPSS 27 software. Experimental data were visualized using GraphPad Prism 8.0 and Origin 2024. Significance levels are indicated as follows: * 0.01 < *p* < 0.05; ** 0.001 < *p* < 0.01; *** 0.0001 < *p* < 0.001.

## 3. Results

### 3.1. Spermidine Attenuates Follicular Atresia and Augments Fertility

**Figure 2:**
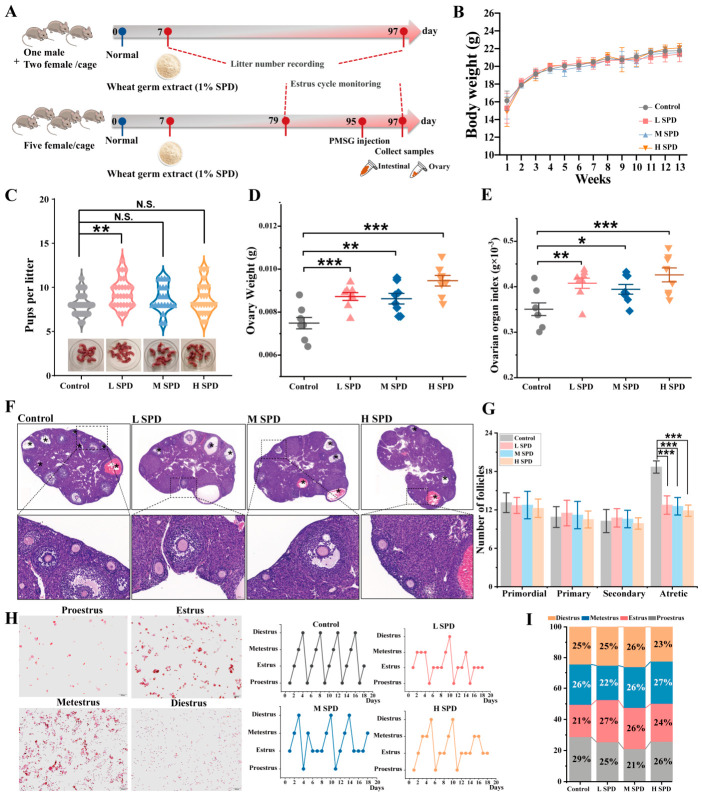
Effects of spermidine on reproductive traits and fertility in mice. (**A**) Timeline of the mouse feeding procedure. (**B**) Cumulative mean weekly body weight changes in each group of mice after spermidine feeding, recorded for 13 weeks (n = 35). (**C**) Representative images of litter size comparison between spermidine-treated mice at different concentrations and control mice. (**D**,**E**) Comparison of ovary weight and organ index among mice in different groups (n = 8). (**F**,**G**) Morphological changes and follicle counts in mouse ovarian tissue slices (n = 4, * atretic follicles). (**H**,**I**) Histogram depicting changes in the estrous cycle and the percentage of each estrous cycle stage (n = 5). SPD: Spermidine; L SPD: 70 mg/kg Spermidine; M SPD: 140 mg/kg Spermidine; H SPD: 210 mg/kg Spermidine; PMSG: Pregnant mare serum gonadotropin. N.S. not significant, * *p* ≤ 0.05, ** *p* ≤ 0.01, *** *p* ≤ 0.001.

The timeline of the feeding process for mice is presented in Figure 2A. Following a 90-day period of dietary spermidine supplementation, no notable variations in body weight were detected with respect to those in the control group (Figure 2B). Fertility experiments demonstrated that feeding mice with spermidine resulted in a significantly greater litter size in the L SPD group (*p* < 0.01; Figure 2C). Additionally, the ovarian organ index significantly increased after feeding (*p* < 0.05; Figure 2D,E). HE staining of ovarian slices revealed no changes in the numbers of primordial follicles, primary follicles, and secondary follicles with spermidine feeding. However, a highly marked reduction in the counts of atretic follicles was observed after spermidine feeding (*p* < 0.001; Figure 2F,G). Furthermore, changes in the estrous cycle in mice were monitored during later stages of feeding. Different concentrations of spermidine led to a significantly longer duration of the average estrous period in mice than that observed in the control group (Figure 2H,I).

### 3.2. Spermidine Promotes the Secretion of Reproduction-Associated Hormones

**Figure 3:**
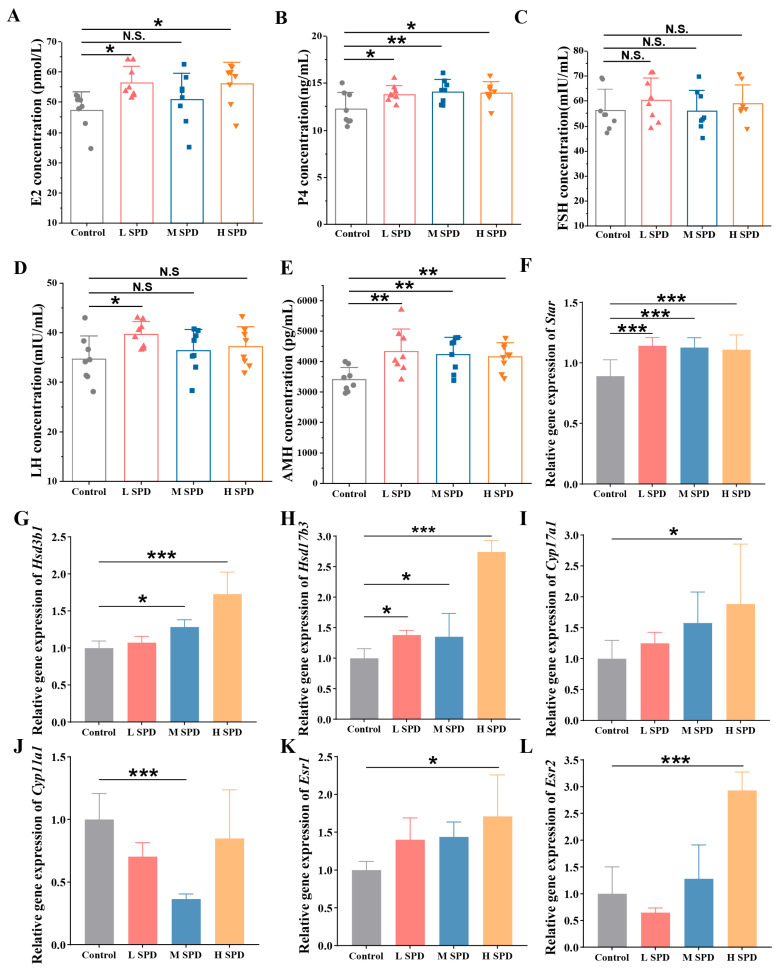
Expression levels of reproductive hormones and their metabolism-related genes. Serum concentrations of E2 (**A**), P4 (**B**), FSH (**C**), LH (**D**), and AMH (**E**) hormones in mice fed different concentrations of spermidine (n = 8). The expression levels of ovarian steroid hormone metabolism-related genes *Star* (**F**), *Esr1* (**G**), *Esr2* (**H**), *Cyp17a1* (**I**), *Cyp11A1* (**J**), *Hsd3b1* (**K**), and *Hsd17b3* (**L**) (n = 6). L SPD: 70 mg/kg Spermidine; M SPD: 140 mg/kg Spermidine; H SPD: 210 mg/kg Spermidine. N.S. not significant, * *p* ≤ 0.05, ** *p* ≤ 0.01, *** *p* ≤ 0.001.

Here, we found that L SPD and H SPD feeding significantly increased the serum E2 levels in mice (*p* < 0.01; Figure 3A). All three concentrations of spermidine significantly elevated serum P4 levels in mice, and M SPD had the most significant effect (*p* < 0.01; Figure 3B). No significant changes in FSH levels were observed among groups (*p* > 0.05; Figure 3C). Moreover, serum LH concentrations showed a marked increase in the L SPD group (*p* < 0.05; Figure 3D) but remained unchanged in the M SPD and H SPD groups (*p* > 0.05; Figure 3D). Notably, AMH levels significantly increased after the spermidine diet, suggesting that spermidine enhances the ovarian reserve capacity in mice (*p* < 0.01; Figure 3E). To further confirm the effect of spermidine on reproductive hormones, we examined the expression levels of genes involved in steroid hormone metabolism. The results showed that spermidine intervention significantly increased the expression of *Star*, a key rate-limiting gene in the initial stage of steroid synthesis (*p* < 0.001; Figure 3F). Except for *Cyp11a1*, the expression levels of key downstream genes in the steroid synthesis pathway, including *Cyp17a1*, *Hsd3b1*, and *Hsd17b3*, were also significantly up-regulated in the H SPD group (*p* < 0.05; Figure 3G–J). Furthermore, the levels of estrogen receptor genes *Esr1* and *Esr2* were notably elevated in the H SPD group (*p* < 0.05; Figure 3K,L), which is consistent with the observed changes in serum steroid hormone levels.

### 3.3. Dietary Spermidine-Rich Feed Increases Polyamine Levels In Vivo

**Figure 4:**
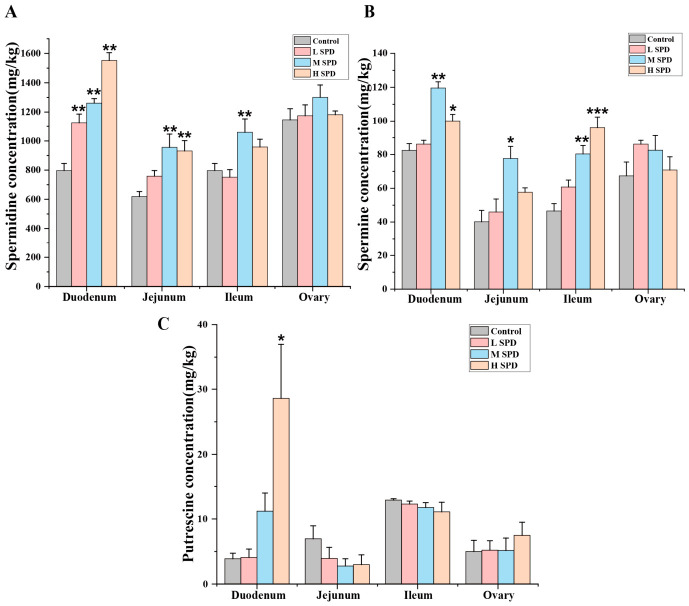
Impact of spermidine on polyamine levels in intestine and ovary. Changes in the content of spermidine (**A**), spermine (**B**), and putrescine (**C**) in the duodenum, jejunum, ileum, and ovaries in mice fed different concentrations of spermidine (n = 6). * *p* ≤ 0.05, ** *p* ≤ 0.01, *** *p* ≤ 0.001. L SPD: 70 mg/kg Spermidine; M SPD: 140 mg/kg Spermidine; H SPD: 210 mg/kg Spermidine.

Analysis of the small intestine, the main site for digestion and absorption of food, can fully reflect nutrient intake. In the present study, we found that all three concentrations of spermidine significantly increased spermidine levels in the duodenum (*p* < 0.01; Figure 4A). In the jejunum, M SPD and H SPD also significantly increased spermidine levels (*p* < 0.01; Figure 4A). In the ileum, spermidine levels were significantly higher in the M SPD group (*p* < 0.01; Figure 4A). With the higher concentrations of spermidine in the M SPD and H SPD groups, more significant effects were observed on the increase in spermine content in all intestinal segments of the small intestine (*p* < 0.05; Figure 4B). Putrescine levels in the small intestine in mice were scarcely affected (*p* > 0.05; Figure 4C), thus suggesting a positive correlation between the amount of polyamine absorbed and the dietary concentration of spermidine, particularly spermidine and spermine. In addition, to clarify whether this absorbed portion of spermidine was directly targeted to the ovarian tissue in mice, we also examined polyamine levels in the ovarian tissue. The absence of changes (Figure 4A–C) suggested that wheat germ-derived spermidine does not exert its effects by directly targeting the ovary. Thus, 16S rRNA and metabolomics sequencing were performed to further explore spermidine’s influence on reproductive potential.

### 3.4. Spermidine Regulates the Levels of Reproduction-Related Serum Metabolites

**Figure 5:**
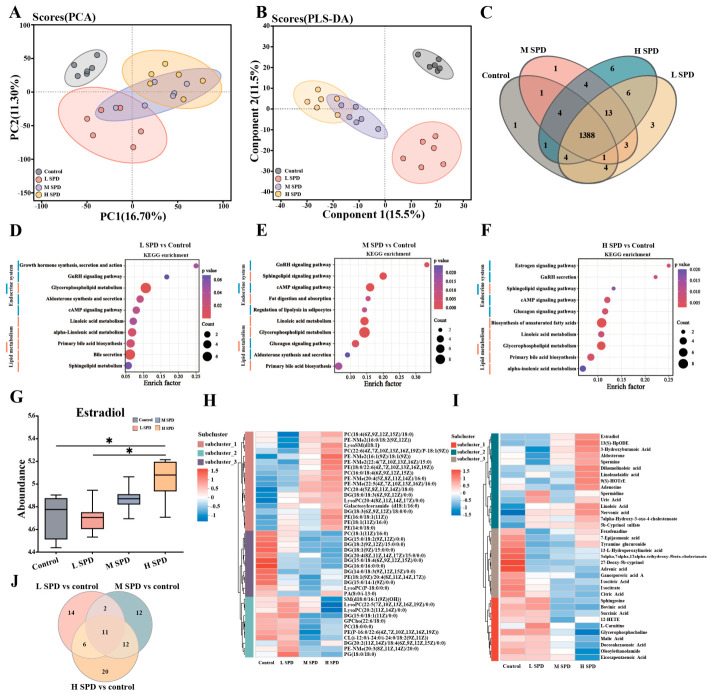
Effects of spermidine on serum reproduction-related metabolite levels. (**A**,**B**) Principal component analysis and PLS-DA assessment of the effects of spermidine on serum metabolic profiles in mice (n = 6). (**C**) Venn diagrams of metabolites among different groups. (**D**–**F**) KEGG enrichment of differential metabolites after comparison of L SPD, M SPD, and H SPD with the control group, respectively. (**G**) Relative expression levels of E2 in metabolome sequencing results. (**H**–**J**) Visualization of the Venn diagram and heat map of differential metabolites enriched in lipid metabolism and associated with the endocrine system. L SPD: 70 mg/kg Spermidine; M SPD: 140 mg/kg Spermidine; H SPD: 210 mg/kg Spermidine. * *p* ≤ 0.05.

The serum metabolic profiles of mice indicated significant changes after dietary spermidine. Dimensionality reduction analysis demonstrated a good separation between the experimental and control groups. Moreover, with feeding of increased concentrations of spermidine, the separation between principal component 1 and principal component 2 became more evident (Figure 5A,B). The Venn diagram indicated that, among the 1440 metabolites detected, 36 were unique to the spermidine feeding group (Figure 5C). Compared with the control group, 312, 275, and 292 differential metabolites were found in the L SPD, M SPD, and H SPD groups, respectively. Through KEGG enrichment analysis, these differential metabolites were found to be enriched primarily in the endocrine system and lipid metabolism-associated pathways (Figure 5D–F). Among them, the GnRH signaling pathway and cAMP signaling pathway, both of which play crucial regulatory roles in animal reproduction, were significantly enriched (Figure 5D–F). Moreover, the differential metabolites in the H SPD group were significantly enriched in the estrogen signaling pathway (Figure 5D–F). In agreement with the experimental results, the concentration of E2 in the metabolomic data also increased with increasing spermidine concentration. Moreover, the E2 concentration in the H SPD group was significantly higher than that in the control group (*p* < 0.05; Figure 5G). The differential expression heatmap presents all differential metabolites associated with the endocrine system and lipid metabolism (Figure 5J). We identified 41 differential metabolites associated with lipid metabolism (Figure 5H) and 36 metabolites associated with the endocrine system (Figure 5I). Collectively, these results illustrated that wheat germ spermidine influenced lipid metabolic processes and enhanced ovarian sex hormone secretion in mice.

### 3.5. Spermidine Induces Changes in Gut Microbiome Composition

**Figure 6:**
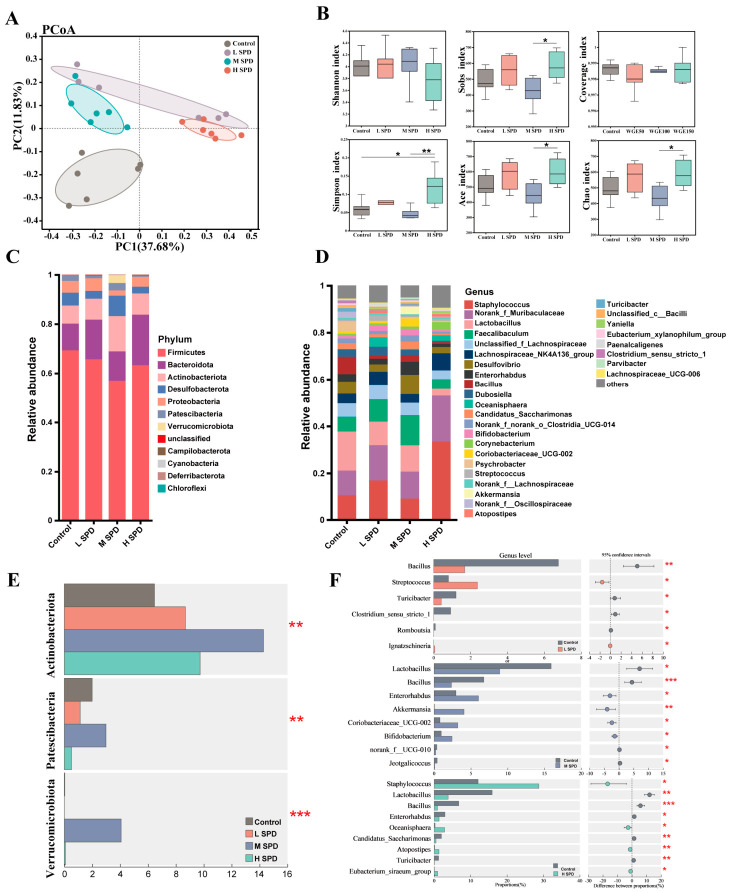
Spermidine induces changes in gut microbiome composition. (**A**) Differences in microbial communities of cecum contents in mice fed spermidine, assessed with PCOA (n = 6). (**B**) Alpha diversity, including the Shannon index, Sobs index, Coverage index, Simpson index, Ace index, and Chao index, used to assess gut microbial diversity among groups. (**C**,**D**) Relative changes in microbial phyla and genera in the cecum after spermidine feeding. (**E**,**F**) Histograms of different phyla and genera of microbes after feeding of different concentrations of spermidine. * *p* ≤ 0.05, ** *p* ≤ 0.01, *** *p* ≤ 0.001.

Optimizing the structure of the gut microbiota is considered an effective strategy to improve animal reproductive performance. Through 16s rRNA sequencing, we observed significant differences in the patterns of changes in the ASV levels of the gut microbiota in the spermidine groups compared with the control group. Additionally, good clustering between groups was observed (Figure 6A). Minimal differences in alpha diversity were observed in mice fed a spermidine diet compared with the controls (Figure 6B). However, significant changes were observed in the gut microbiome composition at both the phylum and genus levels (Figure 6C,D). To visualize the variations in phylum and genus differences among groups, we revealed significant differences in abundance among the phyla *Actinobacteriota*, *Patescibacteria*, and *Verrucomicrobiota*. The abundance of all three phyla was highest in the M SPD group (Figure 6E). The abundance of genera in the L SPD, M SPD, and H SPD groups was compared with that in the control group. We identified six differentially varying genera in the L SPD group, eight differentially varying genera in the M SPD group, and nine differentially varying genera in the H SPD group (Figure 6F). The abundance of *Bacillus* was significantly down-regulated in all these differential genus; the abundance of *Streptococcus* and *Ignatzschineria* was significantly up-regulated in the L SPD group, and the abundance of *Turicibater*, *Clostridiun_sensu_stricto_1*, and *Romboutsia* was significantly down-regulated. The genera *Enterorhabdus*, *Akkermansia*, *Coriobacteriaceae_UCG-002*, and *Bifidobacterium* were significantly up-regulated in the M SPD group, whereas *Lactobacillus*, *norank_f_UGC-010*, and *Jeotgalicoccus* were significantly down-regulated. The genera *Staphylococcus*, *Oceanisphaera*, *Atopostipes*, and *Eubacterium_siraeum_group* were significantly up-regulated in the H SPD group, whereas the genera *Lactobacillus*, *Enterorhabdus*, *Candidatus_Saccharimonas*, and *Turicibacter* were significantly down-regulated (Figure 6F).

### 3.6. Co-Relationship Analysis Between Differential Gut Microbes and Metabolites

**Figure 7:**
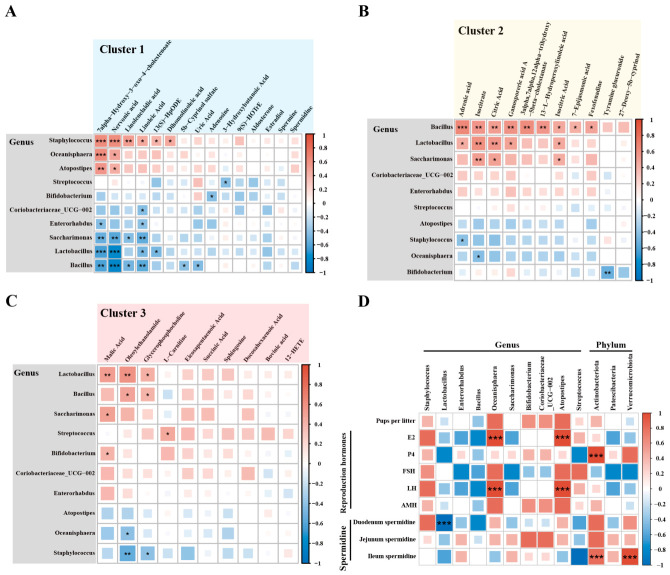
Prediction of the interactions among microbes, metabolites, and indicators of ovarian function in mice. (**A**–**C**) Correlation between differential gut microbial communities and serum hormone secretion-associated differential metabolites: cluster 1 (up-regulated differential metabolites, 15 metabolites), cluster 2 (up-regulated differential metabolites, 11 metabolites), and cluster 3 (up-regulated differential metabolites, 10 metabolites) are from Figure 5I. (**D**) Correlation analysis of litter size, levels of reproduction-associated hormone secretion, and intestinal spermidine levels with differential microbial communities in mice. * *p* ≤ 0.05, ** *p* ≤ 0.01, *** *p* ≤ 0.001.

We excluded genera with low abundance and those with large differences in abundance among samples within a group. Ten genera that were significantly altered by dietary spermidine were retained. Spearman correlation analysis of differential gut microbes and differential metabolites demonstrated that two metabolites, 7-alpha-hydroxy-3-oxo-4-cholestenoate and nervonic acid, in cluster 1 (differentially down-regulated), were significantly positively correlated with *Staphylococcus* and *Oceanisphaera*, and were significantly negatively correlated with *Saccharimonas*, *Bacillus*, and *Lactobacillus* (Figure 7A). *Bacillus*, *Lactobacillus*, and *Saccharimonas* were positively correlated with nine differential metabolites in cluster 2 (differentially up-regulated), such as adrenic acid and isocitrate, whereas *Bifidobacterium* showed a significant negative correlation (Figure 7B). Additionally, three metabolites, malic acid, oleoylethanolamide, and glycerophosphocholine, were positively correlated with *Lactobacillus*, *Bacillus*, and *Saccharimonas* in cluster 3 (down-regulation of differences) and negatively correlated with *Staphylococcus* and *Oceanisphaera* (Figure 7C). Subsequently, we conducted further correlation analysis on litter size, reproduction-associated hormone levels, spermidine levels, and differential microbial communities. Two genera, *Oceanisphaera* and *Atopostipes*, were significantly positively correlated with E2 and LH, and the phylum *Actinobacteriota* was significantly positively correlated with P4. The phyla *Verrucomicrobiota* and *Actinobacteriota* both exhibited significant positive correlations with ileal spermidine levels. In addition, *Lactobacillus* showed a significant negative correlation with duodenal spermidine levels (Figure 7D).

## 4. Discussion

In recent years, numerous studies have reported that various plant-derived foods contain spermidine, with wheat germ showing the highest concentration. Currently, wheat germ represents the most widely used natural source of spermidine commercially available and has been employed in studies examining its anti-inflammatory and anti-aging properties [41]. Notably, as early as 2017, spermidine-enriched wheat germ extract was approved as a novel food by the European Commission. In this study, we used spermidine-enriched wheat germ extract rather than synthetic spermidine. In contrast to the commonly reported acid-based type extraction methods, we applied an ethanol-based extraction protocol, which reduced the levels of unwanted components. Previous comparative research demonstrated that naturally derived spermidine from wheat germ exhibits biological effects comparable to those of synthetic spermidine [53,54]. Furthermore, wheat germ extract contains only trace amounts of other polyamines (spermine and putrescine), eliminating interference from these polyamines and confirming that spermidine is the primary compound.

Previous studies have found that exogenous supplementation with synthesized spermidine promotes oocyte maturation, fertilization, and embryo development, thereby enhancing the fertility of aged mice [10]. In this study, a significant difference was observed in the L SPD group, which showed approximately one or two mice more than observed in the control group. Follicle development is a complex process co-regulated by intrafollicular factors and peripheral hormones, progressing through the stages of primordial, primary, secondary, and antral follicles, and is characterized by the prevalence of follicular atresia [55]. As the physiological basis of female reproductive performance, normal follicular development is essential for maintaining reproductive capacity, whereas follicular atresia leads to a decline in ovarian reserve and impairs fertility [56]. Previous studies have demonstrated that approximately 99% of the follicles in the ovaries experience atresia degeneration, and only a fraction of the follicles reach the pre-ovulatory stage during each reproductive cycle [57,58]. Our ovarian HE staining results showed that wheat germ spermidine significantly decreased the number of atretic follicles under a normal diet in mice; this decrease may be an important reason for the increase in fertility. Furthermore, different concentrations of dietary wheat germ spermidine improved the ovarian organ index in mice. The estrous cycle of mice typically lasts 4–5 days. Therefore, we measured the changes over 18 days (approximately three estrous cycles). Wu et al. reported that 3-nitropropionic acid, an oxidative stress inducer, impairs normal ovarian function and significantly disrupts the estrous cycle in mice [59]. Moreover, our previous study yielded similar results, showing that the estrous cycle in mice was completely disrupted in the presence of a harmful substance [15]. However, not all changes in the estrous cycle are detrimental; a moderate prolongation of the estrous period throughout the mammalian estrous cycle can increase ovulation and fertility [60]. Although the molecular mechanisms linking the estrous cycle to reproductive regulation remain unclear, numerous studies have confirmed the beneficial effects of nutrient supplementation on the induction of estrus and reproductive performance in animals [61,62]. In contrast, severe nutrient deficiency, such as protein deficiency, can lead to arrest of the estrous cycle or impaired follicular development [63]. In our study, we found that wheat germ-derived spermidine extended the average duration of the estrous period in mice. Notably, this change was distinct from pathological estrous cycle disruption, as it was characterized by a regular prolongation of the estrous period. These findings suggest that the prolongation of the estrous period induced by wheat germ-derived spermidine in mice may be associated with improved fertility. Subsequently, the results for serum reproduction-associated hormones further explained the changes in fertility, and E2 was significantly up-regulated in both the L SPD and H SPD groups, whereas P4 showed a tendency toward up-regulation in each treatment group. Changes in hormone levels were consistent with findings in avian species, suggesting that spermidine promotes the secretion of estrogen and progesterone across different species [64]. To further assess alterations in steroid hormone levels, we examined the expression of genes involved in the steroid hormone synthesis pathway as well as estrogen receptors. The results indicated that spermidine treatment significantly upregulated *Star*, a key regulatory gene controlling the initial step of steroidogenesis [65]. Moreover, the expression of major downstream steroidogenic genes, such as *Cyp17a1*, *Hsd3b1*, and *Hsd17b3*, was markedly increased. These findings suggest activation of the steroidogenic pathway, which may in turn promote the production of downstream sex hormones [66], a result consistent with the aforementioned function of spermidine in promoting hormone secretion. Consistent with these findings, the expression levels of estrogen receptors ESR1 and ESR2 were also significantly elevated. These results suggested an enhanced effect of wheat germ spermidine on the secretion of ovarian steroid hormones in mice. Serum AMH levels are commonly used for assessing the functional ovarian reserve. AMH is secreted by functional granulosa cells in developing antral and small sinus follicles, and its expression is absent in atretic follicles and the corpus luteum [67]. Corresponding to the number of atretic follicles, serum AMH levels significantly increased after wheat germ spermidine feeding, and this effect was not affected by the concentration of wheat germ spermidine, thus suggesting that ovarian AMH secretion is highly sensitive to wheat germ spermidine.

Dietary supplementation with spermidine is considered an effective strategy for coping with the decline in spermidine concentrations with aging [68,69,70]. To clarify whether wheat germ spermidine might be sufficiently absorbed and might act by directly targeting the ovaries, we examined the polyamine content in small intestinal and ovarian tissues. As expected, the intestinal spermidine content significantly increased after spermidine feeding, in a dose-dependent manner. Therefore, dietary wheat germ spermidine can be successfully absorbed and used by the intestines. Moreover, beyond spermidine, we observed changes, albeit less pronounced than those in spermidine, in the content of the other two polyamines, spermine and putrescine. These findings suggested that wheat germ spermidine supplementation affects the metabolic levels of intestinal polyamines and increases polyamine production. However, we did not find that spermidine targeted the ovaries to exert its effects, as reported in previous studies. Following spermidine supplementation, the polyamine content in ovarian tissues remained unchanged. This indicates that spermidine does not exert its effects by acting directly on the ovary but may instead improve reproductive capacity by modulating gut microbial metabolism.

Serum metabolomics revealed significant changes in serum metabolite profiles after wheat germ spermidine diet feeding. The extent of changes in serum metabolite profiles positively correlated with changes in wheat germ spermidine concentrations. In addition, 36 new metabolites were detected after wheat germ spermidine diet feeding compared with control. KEGG enrichment analysis was performed on these differential metabolites, which were enriched primarily in pathways associated with lipid metabolism and the endocrine system. Among them, both the GnRH signaling pathway and the cAMP signaling pathway in the endocrine system are closely associated with animal reproduction [71,72,73]. Hypothalamic gonadotropin-releasing hormone (GnRH), a crucial regulator of the reproductive system, modulates the synthesis and release of LH and FSH in the pituitary gland, and controls gametogenesis and steroidogenesis [74]. Intracellular cAMP accumulation leads to activation of protein kinase A (PKA). Subsequently, PKA triggers the phosphorylation of the transcription factor cAMP-responsive element binding protein (CREB) and extracellular-regulated kinase (ERK1/2) [71]. In steroid-secreting cells, the activation of phospho-proteins is associated with the expression of target genes, which in turn is typically associated with cholesterol transport into the mitochondria and the synthesis of sex hormones [75]. Moreover, cAMP signaling is an important signal transduction pathway through which LH and FSH exert their effects within follicle [76,77]. In addition, Cai et al. found that cAMP, an important intracellular secondary messenger, regulates the downstream PKA-CREB pathway, thereby decreasing ovarian granulosa cell apoptosis and protecting ovarian function [78]. Beyond these two pathways, the H SPD group showed significant enrichment in differential metabolites in the estrogen signaling pathway. Subsequently, estradiol was visualized in the serum metabolic profiling results. In agreement with the experimental results, the estradiol content increased with rising concentrations of dietary wheat germ spermidine, and estradiol levels were significantly higher in the H SPD group compared with control. This finding further confirmed that wheat germ spermidine is involved in regulating ovarian hormone secretion and the reproductive process in mice.

Gut microbes influence distant organs and pathways, and are considered a mature endocrine organ [39]. Through interactions with estrogen, androgens, insulin, and other hormones, gut microbes play important roles in the endocrine system throughout the life of female animals [39,79,80]. Sex hormones such as E2, P4, and testosterone are also involved in microbe-host communication and play important physiological roles in animal reproduction [39], as evidenced by antibiotic use leading to lower estrogen levels [81]. On this basis, we hypothesized that the ovarian-improving function of wheat germ spermidine was due to variations in the structure of the gut microbiota. To further confirm this hypothesis, we performed 16s rRNA sequencing of the cecum contents. After a wheat germ spermidine diet, we observed no significant changes in gut microbial diversity, but we identified significant differences in the structural composition of the gut microbes between the spermidine group and the control group. We identified three differentiated phyla and 18 differentiated genera through differential analysis. Differential microbial-metabolite correlation analysis revealed that two metabolites, 7-alpha-hydroxy-3-oxo-4-cholestenoate and nervonic acid, were significantly positively correlated with *Staphylococcus* and *Oceanisphaera*, and significantly negatively correlated with *Saccharimonas*, *Bacillus*, and *Lactobacillus*. *Bacillus*, *Lactobacillus*, and *Saccharimonas* were positively correlated with nine differential metabolites in cluster 2, and *Bifidobacterium* was significantly negatively correlated with tyramine glucuronide. Three metabolites, malic acid, oleoylethanolamide, and glycerophosphocholine, were positively correlated with *Lactobacillus*, *Bacillus*, and *Saccharimonas*, and negatively correlated with *Staphylococcus* and *Oceanisphaera*. These results correlate the effects of wheat germ spermidine on intestinal communities and serum metabolism, and might potentially explain the improvements in ovarian function. To investigate the relationships of these gut microbial communities with polyamine levels and hormone secretion, we performed further correlation analysis. The interaction between sex hormones and the gut microbiome plays an important role in the development of metabolic diseases [82]. E2 and P4 levels have been found to change significantly after microbial colonization in mice [83]. Furthermore, Org et al. used gonadectomy to demonstrate that sex hormones significantly influence the composition of the gut microbiota, and have observed higher microbial diversity in individuals with elevated serum E2 levels [84]. Additionally, two bacteria, *Slackia* and *Butyricimonas*, have been correlated with E2 levels [85]. In the present study, both Oceanisphaera and Atopostipes were significantly positively correlated with E2 and LH. The phylum *Actinobacteriota* was significantly positively correlated with P4. The genera *Verrucomicrobiota* and *Actinobacteriota* were both significantly positively correlated with ileal spermidine levels. In addition, *Lactobacillus* showed a significant negative correlation with duodenal spermidine content. The correlation analysis results predicted the interactions between gut microbes and metabolites. However, further in vivo validation of the functions of specific strains is required. In this study, we only preliminarily explored the effects through which wheat germ-derived spermidine improves reproductive capacity in mice, with an aim of providing new ideas and insights for subsequent studies in animal reproduction.

## 5. Conclusions

**Figure 8:**
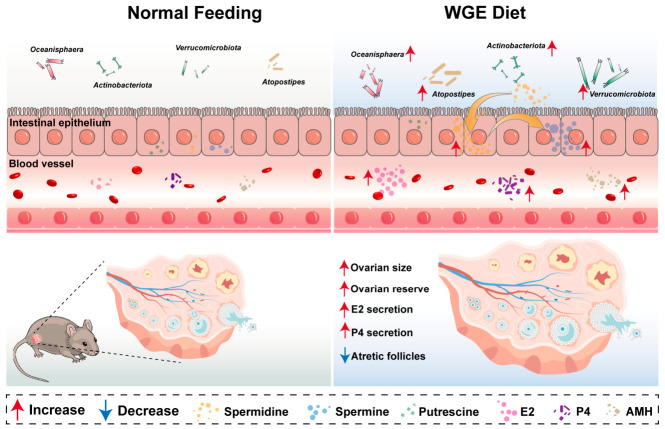
Mechanism by which spermidine improves ovarian function in mice through mediating gut microbes (diagram with Adobe Illustrator 2023). WGE: Wheat germ extract; E2: Estradiol; P4: Progesterone; AMH: Anti-Mullerian hormone.

Collectively, our results reveal that dietary spermidine increased polyamine levels in intestinal tissue and affected the metabolism of spermine and putrescine. Spermidine decreased the number of atretic follicles and improved ovarian function, including E2 and P4 secretion as well as the ovarian reserve capacity of the mice, thereby enhancing fertility. Furthermore, the structural changes in the intestinal microbiota were closely associated with changes in reproduction-associated hormone levels. Two genera, Oceanisphaera and Atopostipes, showed significant positive correlations with E2 and LH, and Actinobacteriota showed a significant positive correlation with P4. These results demonstrate that spermidine modulates the composition of gut microbiota and metabolites, thereby enhancing steroidogenesis and influencing fertility in mice (Figure 8). These discoveries broaden the research scope of spermidine and offer several phenotypic insights into the role of spermidine in enhancing reproductive performance in mammals.

## Figures and Tables

**Table 1:** Nutritional composition of mouse maintenance and breeding feed.

Testing Items	Testing Methods	Maintenance Feed (g/kg)	Breeding Feed (g/kg)
Water	GB/T6435-2014 8.1 [46]	94	95
Protein	GB/T6432-2018 7.2 [47]	185	205
Fat	GB/T6433-2006 9.3 [48]	52	56
Fibre	GB/T6434-2006 [49]	35	33
Ash	GB/T6438-2007 [50]	66	68
Calcium	GB/T6436-2018 [51]	11.3	12.7
Phosphorus	GB/T6437-2018 [52]	8.6	8.5

Note: Wheat germ extract containing 1% spermidine was added to the basal diet at 50, 100, and 150 g/kg during feed mixing, and all determinations were performed in accordance with Chinese national recommended standards (GB/T).

**Table 2:** RT-qPCR primer sequence information.

Genes	Primer Sequence (5′-3′)	Amplicon Size (bp)	Accession No.
*Star*	F: CTTGGCTGCTCAGTATTGAC	153	NM_011485.5
	R: TGGTGGACAGTCCTTAACAC		
*Cyp11a1*	F: GATCCCGAGGCCCAGCGGTT	323	NM_001346787.1
	R: AGGGTCATGGAGGTCGTGTCCA		
*Cyp17a1*	F: GCCCCAGATGGTGACTCAAAGCC	569	NM_007809.3
	R: ACACATCTGGGTCCCGGCCT		
*Hsd3b1*	F: GCCCCTGATCTTTTCAGCCACCAC	311	XM_006501036.3
	R: GGGTAACCCTTAGGAGGGCTGTTAA		
*Hsd17b3*	F: TGGAGTCAAGGAGGAAAGGCCTCA	366	NM_008291.3
	R: GGAATCGTTGAGCGGTGCTGC		
*Esr1*	F: CCAGCAGTAACGAGAAAGGAAAC	95	NM_007956.6
	R: ATAATGGTAGCCAGAGGCATAGTC		
*Esr2*	F: GACGAAGAGTGCTGTCCCAA	209	NM_207707.1
	R: TCAGCTTCCGGCTACTCTCT		
*β-actin*	F: AGAAGATCTGGCACCACACC	172	NM_177093.3
	R: TACGACCAGAGGCATACAGG		

Note: *Star*, Steroidogenic Acute Regulatory Protein; *Cyp11a1*, Cytochrome P450 Family 11 Subfamily A Member 1; *Cyp17a1*, Cytochrome P450 Family 17 Subfamily A Member 1; *Hsd3b1*, Hydroxy-delta-5-steroid Dehydrogenase, 3 beta- and Steroid Delta-isomerase 1; *Hsd17b3*, Hydroxysteroid (17-beta) Dehydrogenase 3; *Esr1*, Estrogen Receptor 1; *Esr2*, Estrogen Receptor 2; *β-actin*, Actin Beta.

## Data Availability

The raw sequence data of 16S rRNA gene sequencing utilized in this study are publicly accessible at https://www.ncbi.nlm.nih.gov/sra/?term=PRJNA1163073 (accessed on 2 June 2026). Additionally, the untargeted metabolomic data used in this study have been deposited in the EMBL-EBI MetaboLights database with the identifier MTBLS11115. The complete dataset can be accessed at https://www.ebi.ac.uk/metabolights/MTBLS11115 (accessed on 2 June 2026).

## Abbreviations

The following abbreviations are used in this manuscript:

E2	Estradiol
P4	Progesterone
FSH	Follicle-stimulating hormone
LH	Luteinizing hormone
AMH	Anti-Mullerian hormone
PMSG	Pregnant mare serum gonadotropin
ELISA	Enzyme-linked immunosorbent assay
HPLC	High-performance liquid chromatography
HE	Hematoxylin–eosin staining
PCR	Polymerase chain reaction
PCA	Principal component analysis
PLS-DA	Partial least squares discriminant analysis
PCoA	Principal coordinate analysis
ASV	Amplicon sequence variant
KEGG	Kyoto encyclopedia of genes and genomes
